# *Nascer no Brasil* II: findings and implications for the State of Rio de Janeiro

**DOI:** 10.11606/s1518-8787.2025059supl1ed

**Published:** 2025-10-20

**Authors:** João Luiz Bastos, Eleonora d’Orsi, Marly Augusto Cardoso, Tonantzin Ribeiro Gonçalves

**Affiliations:** I Simon Fraser University. Faculty of Health Sciences. Burnaby, BC, Canada; II Universidade Federal de Santa Catarina. Departamento de Saúde Pública. Florianópolis, SC, Brasil; III Universidade de São Paulo. Faculdade de Saúde Pública. Departamento de Nutrição. São Paulo, SP, Brasil; IV Universidade Federal de Ciências da Saúde de Porto Alegre. Departamento de Psicologia. Porto Alegre, RS, Brasil

Conducted with the aim of updating and expanding the data from the *Nascer no Brasil I* Survey (2011–2012)^1^, the *Nascer no Brasil II* study (2021–2023)^2^ incorporated new and highly relevant topics, including abortion, paternal mental health, and obstetric violence, among others. In addition to these thematic advancements, the study expanded its sample in the state of Rio de Janeiro, enabling the production of representative estimates for this federative unit — an achievement not yet attained for other Brazilian states. Taking advantage of this analytical opportunity, this special supplement of the *Revista de Saúde Pública* presents seven articles that comprehensively examine key aspects of maternal and child health in the context of Rio de Janeiro.

The published articles address a broad range of topics, including:

- effects of extreme maternal ages on maternal and perinatal outcomes;
- obstetric violence;
- postpartum mental disorders;
- adequacy of prenatal care;
- hospital practices related to breastfeeding;
- inequalities in childbirth care;
- use of the care network according to gestational risk.

Grounded in quantitative methodologies and probabilistic sampling, the studies offer a detailed overview of the key challenges faced by women during pregnancy, childbirth, and the postpartum period in the state of Rio de Janeiro. The findings are essential for monitoring health inequities, informing public policy, and enhancing the quality of health care.

The article by Silvana da Gama et al. demonstrates that maternal age directly influences both the care received and clinical outcomes. Women under the age of 20 experienced more precarious prenatal and childbirth care, whereas those aged 35 and older showed a higher prevalence of conditions such as hypertensive syndromes, gestational diabetes, and severe maternal morbidity. These findings highlight the need for differentiated care strategies tailored to the specific characteristics of each age group.

Obstetric violence and psychological distress related to childbirth and the postpartum period are addressed in two additional studies. Tatiana Leite et al. identified an overall prevalence of obstetric violence at 65.3%, with the most frequently reported practices being inappropriate vaginal examinations (46.2%), followed by neglect (31.5%) and psychological abuse (21.7%). These forms of mistreatment disproportionately affect women experiencing social vulnerability. In addition to these findings, the study by Mariza Theme-Filha et al. reported concerning rates of postpartum symptoms of depression (17.9%), anxiety (16.3%), and post-traumatic stress disorder (7.7%), particularly among women with lower levels of education. Together, the studies depict a scenario of psychological distress that intensifies existing inequalities in reproductive health. Preventing the perpetuation of violence within hospital settings and strengthening mental health care throughout the prenatal and postpartum periods emerge as critical areas for public policy investment.

The analysis conducted by Rosa Domingues and her team underscores the low adequacy of prenatal care in the state, despite relatively favorable indicators for early initiation (78.8%) and the minimum number of consultations (75.5%). When considering the full set of components recommended by the World Health Organization and the Brazilian Ministry of Health — such as laboratory testing, immunization, supplementation, and health education —, fewer than 1% of pregnant women received fully adequate care. The primary shortcoming, therefore, lies in the quality of care, particularly among women experiencing social vulnerability.

Complementing this overview, the study led by Ana Paula Esteves-Pereira examined the relationship between hospital practices and breastfeeding. While breastfeeding was nearly universal within the first 24 hours of life, the rate of exclusive breastfeeding dropped to 61.4% by two months of age, with higher prevalence among mothers with greater educational attainment and those who gave birth in facilities affiliated with the Baby-Friendly Hospital Initiative. The early introduction of pre-lacteal foods was associated with a decline in exclusive breastfeeding during the initial months of life. These findings underscore the need to strengthen efforts to promote, protect, and provide institutional support for breastfeeding, particularly in socially vulnerable contexts.

Two articles focus on the organization of services and the adequacy of care based on gestational risk. Maria do Carmo Leal et al. reported a low prevalence of labor (54.4%) and vaginal delivery (41%) in the state. The study by Sonia Bittencourt et al. found that nearly one-third (30.5%) of high-risk pregnant women received care in maternity hospitals lacking maternal or neonatal intensive care units, revealing significant deficiencies in resource allocation and care regulation. Both studies identified critical issues that demand urgent action and sustained investment.

Taken together, the articles in this supplement make a significant contribution to advancing a strategic agenda for maternal and child health in the state of Rio de Janeiro. While acknowledging important progress, the findings highlight ongoing challenges in delivering qualified, equitable, and woman- and newborn-centered care. The *Revista de Saúde Pública* is committed to supporting the dissemination of this evidence and fostering scientific dialogue on strategies to address and overcome health inequities. Enjoy your reading!
