# Supplementary material for: Nascer no Brasil II: findings and implications for the State of Rio de Janeiro
Source: Rev Saude Publica. 2025 Oct 20;59(Suppl 1):e1s. doi: 10.11606/s1518-8787.2025059supl1ed (PMC12539632; doi:10.11606/s1518-8787.2025059supl1ed)
Supplement: Translation

# Translation: Nascer no Brasil II: achados e implicações para o estado do Rio de Janeiro

**Authors:** João Luiz Bastos, Eleonora d’Orsi, Marly Augusto Cardoso, Tonantzin Ribeiro Gonçalves

**Affiliations:** I Simon Fraser University. Faculty of Health Sciences. Burnaby, BC, Canada; II Universidade Federal de Santa Catarina. Departamento de Saúde Pública. Florianópolis, SC, Brasil; III Universidade de São Paulo. Faculdade de Saúde Pública. Departamento de Nutrição. São Paulo, SP, Brasil; IV Universidade Federal de Ciências da Saúde de Porto Alegre. Departamento de Psicologia. Porto Alegre, RS, Brasil

Elaborado com os objetivos de atualizar e ampliar os dados obtidos na Pesquisa Nascer no Brasil I (2011–2012)^1^, o estudo Nascer no Brasil II (2021–2023)^2^ incorporou novos temas de grande relevância, como aborto, saúde mental paterna e violência obstétrica, entre outros. Além do avanço temático, a nova edição contou com uma ampliação da amostra no estado do Rio de Janeiro, o que permitiu gerar estimativas representativas para essa unidade da federação — uma possibilidade ainda ausente para os demais estados brasileiros. Com base nessa oportunidade analítica, este suplemento especial da *Revista de Saúde Pública* reúne sete artigos que exploram, de forma robusta, aspectos centrais da saúde materno-infantil no contexto fluminense.

Os trabalhos publicados cobrem uma ampla gama de temas, que incluem:

- efeitos de extremos etários sobre desfechos maternos e perinatais;
- violência obstétrica;
- transtornos mentais no pós-parto;
- adequação do pré-natal;
- práticas hospitalares relacionadas ao aleitamento materno;
- desigualdades na atenção ao parto;
- utilização da rede de cuidados conforme o risco gestacional.

Com base em metodologias quantitativas e amostragem probabilística, os estudos oferecem um panorama detalhado dos principais desafios enfrentados pelas mulheres durante a gestação, o parto e o puerpério, no estado do Rio de Janeiro. Seus achados são fundamentais para o monitoramento de iniquidades, a formulação de políticas públicas e o aprimoramento da qualidade da atenção à saúde.

No artigo assinado por Silvana da Gama et al., evidencia-se como a idade materna influencia diretamente a assistência recebida e os desfechos clínicos. Mulheres com menos de 20 anos enfrentaram maior precariedade no atendimento pré-natal e no parto, enquanto aquelas com mais de 35 anos apresentaram maior prevalência de condições como síndromes hipertensivas, diabetes gestacional e morbidade materna grave. Esses resultados indicam a necessidade de estratégias assistenciais diferenciadas ajustadas às especificidades de cada grupo etário.

A violência obstétrica e o sofrimento psíquico relacionado ao parto e ao puerpério são temas de dois outros estudos. Tatiana Leite et al. identificaram prevalência geral de 65,3% de violência obstétrica, com destaque para os toques vaginais inadequados (46,2%), seguidos por negligência (31,5%) e abuso psicológico (21,7%). Tais práticas incidem de forma desproporcional sobre mulheres em situação de vulnerabilidade social. Associando-se a essas experiências, o estudo de Mariza Theme-Filha et al. revelou frequências preocupantes de sintomas de depressão (17,9%), ansiedade (16,3%) e transtorno de estresse pós-traumático (7,7%) no pós-parto, especialmente entre mulheres com menor escolaridade. Juntos, os trabalhos revelam um cenário de sofrimento psíquico que agrava as desigualdades já existentes no campo da saúde reprodutiva. Desse modo, tanto evitar a reprodução de violências no contexto hospitalar quanto fortalecer a atenção em saúde mental no cuidado durante o pré-natal e puerpério são pontos de investimento das políticas públicas.

A análise conduzida por Rosa Domingues e sua equipe destaca a baixa adequação do pré-natal no estado, mesmo com indicadores relativamente satisfatórios de início precoce (78,8%) e número mínimo de consultas (75,5%). Considerando o conjunto de componentes recomendados pelas diretrizes da Organização Mundial da Saúde e do Ministério da Saúde — como exames laboratoriais, imunização, suplementação e orientações —, menos de 1% das gestantes receberam atenção plenamente adequada. A principal lacuna reside, portanto, na qualidade do cuidado, especialmente entre mulheres socialmente mais vulneráveis.

Complementando esse panorama, o estudo liderado por Ana Paula Esteves-Pereira investigou o vínculo entre práticas hospitalares e aleitamento materno. A amamentação foi quase universal nas primeiras 24 horas de vida, mas o aleitamento exclusivo aos dois meses diminuiu para 61,4%, com melhores taxas entre mães de maior escolaridade e que deram à luz em instituições associadas à Iniciativa Hospital Amigo da Criança. A introdução de alimentos pré-lácteos esteve associada à redução da amamentação exclusiva nos primeiros meses de vida. Esses achados reforçam a importância de intensificar-se as ações para promoção, proteção e apoio institucional à prática do aleitamento, especialmente em contextos de maior vulnerabilidade social.

Dois artigos abordam a organização dos serviços e a adequação da assistência conforme o risco gestacional. Maria do Carmo Leal et al. apontam a baixa prevalência de trabalho de parto (54,4%) e de parto vaginal (41%) no estado. Já o estudo de Sonia Bittencourt et al. revelou que quase um terço (30,5%) das gestantes de alto risco foi atendido em maternidades sem unidades de terapia intensiva materna ou neonatal, evidenciando falhas graves na alocação de recursos e na regulação do cuidado. Ambos os estudos evidenciaram pontos críticos que exigem ação urgente e investimento contínuo.

Em seu conjunto, os artigos reunidos neste suplemento contribuem de forma significativa para o fortalecimento de uma agenda estratégica em saúde materno-infantil no estado do Rio de Janeiro. Embora os estudos reconheçam avanços importantes, os dados apontam para persistentes desafios na oferta de atenção qualificada, equitativa e centrada nas necessidades das mulheres e dos recém-nascidos. A *Revista de Saúde Pública* tem orgulho de colaborar com a divulgação dessas evidências e de fomentar o debate científico sobre caminhos possíveis para a superação das iniquidades. Boa leitura!
